# Adding salt to foods and risk of metabolic dysfunction-associated steatotic liver disease and other chronic liver diseases

**DOI:** 10.1007/s00394-025-03745-3

**Published:** 2025-06-21

**Authors:** Shunming Zhang, Zhenyu Huo, Yan Borné, Emily Sonestedt, Lu Qi

**Affiliations:** 1 School of Public Health, Xi’an Jiaotong University Health Science Center, Xi’an, Shaanxi China; 2 Nutritional Epidemiology, Department of Clinical Sciences Malmö, Lund University, Malmö, Sweden; 3 School of Public Health, North China University of Science and Technology, Tangshan, Hebei,, China; 4 Department of Epidemiology, Celia Scott Weatherhead School of Public Health and Tropical Medicine, Tulane University, New Orleans, LA USA; 5 Department of Nutrition, Harvard T.H. Chan School of Public Health, Boston, MA USA

**Keywords:** Salt, Sodium, Metabolic dysfunction-associated steatotic liver disease, Cirrhosis, Hepatocellular carcinoma

## Abstract

**Purpose:**

Adding salt to foods, a novel indicator for studying habitual sodium intake, has been positively associated with multiple diseases and mortality. However, little is known about its association with liver-related disorders. This study aimed to investigate the associations of adding salt to foods with risks of metabolic dysfunction-associated steatotic liver disease (MASLD), cirrhosis, and hepatocellular carcinoma (HCC).

**Methods:**

This prospective cohort study included 492,265 participants from the UK Biobank without prevalent liver diseases at baseline. The frequency of adding salt to foods was collected using a self-reported question, and incident liver-related disorders were identified through electronic health records. Multivariable Cox proportional hazard models were used to estimate hazard ratios (HRs) and 95% confidence intervals (CIs) for the outcomes.

**Results:**

Over a median follow-up of 13 years, 7,005 incident MASLD cases, 5,546 cirrhosis cases, and 413 HCC cases occurred. After adjusting for sociodemographic characteristics, lifestyle factors, personal history of diseases, and diet factors, the HRs (95% CIs) of MASLD across the increasing frequency of adding salt to foods were 1.00 (reference) for never/rarely, 1.08 (1.02, 1.14) for sometimes, 1.22 (1.13, 1.31) for usually, and 1.40 (1.27, 1.53) for always, with a *P* for trend < 0.0001. Such association was partly driven by adiposity. Also, similar positive associations were observed for cirrhosis and HCC.

**Conclusions:**

A higher frequency of adding salt to foods was associated with increased risks of MASLD, cirrhosis, and HCC. Reducing adding salt to foods at the table could be included in public health initiatives to promote liver health.

**Supplementary Information:**

The online version contains supplementary material available at 10.1007/s00394-025-03745-3.

## Introduction

Metabolic dysfunction-associated steatotic liver disease (MASLD), formerly known as non-alcoholic fatty liver disease, is a growing public health challenge, currently affecting around 30% of the global population [1]. Of concern, this figure is projected to rise to 55.7% by 2040 [2]. People with MASLD not only can progress to cirrhosis (globally the 9th leading cause of death in Europe [3]) and hepatocellular carcinoma (HCC, ranking as the third leading cause of cancer-related mortality [4]), but also are particularly at increased risk of extrahepatic complications such as cardiovascular disease (CVD), type 2 diabetes, chronic kidney disease, and extrahepatic cancers [5]. Nevertheless, only one drug (resmetirom) is currently approved for MASLD [6]. Therefore, prevention and early detection of modifiable risk factors of MASLD as well as cirrhosis and HCC are still a high public health priority.

Sodium is an important dietary component and serves many physiological functions such as nutrient absorption and maintaining fluid balance [7]. There has been a growing interest in sodium intake owing to its wide-range healthy relevance. Several cross-sectional studies have examined the association of dietary sodium intake assessed by dietary survey or urinary sodium excretion (most based on spot urine and only one collected two complete consecutive 24-hour urine) with MASLD, with reporting a positive association [8–14] or null association [15]. These studies were limited to drawing causal conclusions due to their study design and inaccurate exposure measurement. In addition, a recent prospective cohort study showed a null association between estimated 24-hour urinary sodium excretion from spot urine specimens and risk of MASLD in males [16]. Of note, sodium intake amount varies widely from day to day. Thus, using sodium measured by dietary survey or single urine sample as a tool to assess long-term sodium intake amounts is inadequate. In this regard, we [17–20] and others [21] recently identified the frequency of adding salt to foods as a new indicator for studying habitual sodium intake and health outcomes in Western countries. Salt added to foods is mainly sodium chloride, thus it can minimize the effect of concomitant factors in the diet, such as potassium and others, which could affect the accuracy of estimating sodium intake. Previous studies have indicated the positive associations between salt added to foods and various adverse health outcomes such as type 2 diabetes [20, 22], CVD [18, 23, 24], and mortality [17], yet the role of added salt in liver-related disorders remains underexplored. This highlights the need for further investigation into the potential impact of habitual sodium consumption on liver health.

Therefore, in the present study, we aimed to assess the hypothesis that a higher frequency of adding salt to foods was associated with higher risks of MASLD, cirrhosis, and HCC. Furthermore, based on our [20] and others’ study [11], we tested whether adiposity mediated the associations. Moreover, magnetic resonance imaging determined liver proton density fat fraction (PDFF) is an accurate and reliable measure of liver fat content over the entire liver [25, 26]; thus, we also investigated the association between adding salt to foods and MASLD defined by PDFF.

## Methods

### Study population

This prospective cohort study participants were from the UK Biobank, initiated between 2006 and 2010 and comprising over 500,000 individuals aged 37–73 years at baseline [27]. During recruitment, data on sociodemographic characteristics, lifestyle factors, and health-related conditions were collected through touchscreen questionnaires, interviews, and physical examinations. The study protocol was approved by the North West Multi-center Research Ethics Committee (REC reference: 11/NW/0382), and electronically signed consent was obtained from all participants. In the primary analyses, we excluded participants who had missing data on baseline age or the frequency of adding salt to foods, were lost to follow-up, or had liver diseases or alcohol/drug use disorders (diagnoses codes are shown in Supplementary Table 1) at baseline [28]; the analytic sample included 492,265 participants (Supplementary Fig. 1). The manuscript was prepared following the Strengthening the Reporting of Observational Studies in Epidemiology (STROBE) guidelines [29] and the STROBE-nut guidelines [30].

### Exposure assessment

Data about the frequency of adding salt to foods were collected by a touchscreen question “Do you add salt to your food? (Do not include salt used in cooking)”, with the response options being “Never/rarely”, “Sometimes”, “Usually”, “Always”, and “Prefer not to answer”. Participants with the response “Prefer not to answer” were assigned as missing values, consistent with previous studies [17, 18, 20].

Urinary sodium and potassium concentrations were measured by the Ion Selective Electrode method using Beckman Coulter AU5400 in random urinary spot samples collected at baseline. Consistent with previous studies [17, 31], the 24-hour sodium excretion (g/day) was estimated from the spot urinary concentration values based on the sex-specific INTERSALT equations. A total of 471,427 participants had available data on urinary sodium concentrations (Supplementary Fig. 1).

### Outcome assessment and follow-up

Incident MASLD was the primary outcome and was defined using the International Classification of Disease, Tenth Revision (ICD-10) codes K76.0 and K75.8 [28]. Secondary outcomes included cirrhosis (ICD-10 codes: K74.0, K74.1, K74.2, K74.6, K76.6, K76.7, R18, I85.0, I85.9, I86.4, I98.2, I98.3) and HCC (ICD-10 code: C22.0). A detailed list of ICD-10 codes and reference papers can be found in Supplementary Table 1. Participants were followed up from the date of completion of the baseline assessment to the date of the outcomes, date of death, or the last date of follow-up (December 19, 2022), whichever came first.

Liver PDFF was measured by magnetic resonance imaging in a small proportion of the participants between 2014 and 2020 [26, 32] (*n* = 40,491, Supplementary Fig. 1), and the threshold for defining MASLD was PDFF ≥ 5% [33].

### Assessment of covariates and potential effect mediators

Potential confounders and effect mediators were identified by the directed acyclic graph approach (Supplementary Fig. 2) [34]. A touchscreen questionnaire was used to collect baseline information on age, sex, ethnicity, Townsend deprivation index (a higher score indicates a lower socioeconomic status), smoking status, alcohol drinking status, alcohol intake, physical activity (calculated as total metabolic equivalent task [MET] minutes per week for walking, moderate and vigorous activity), and sedentary time. Prevalent CVD and cancer were identified by self-reported disease history and ICD-10 codes. Diabetes was defined by self-reported disease history, use of hypoglycemic medications, ICD-10 codes E10 to E14, and/or glycated hemoglobin of ≥ 48 mmol/L. Hyperlipidemia was defined as taking cholesterol-lowering medications, triglycerides ≥ 1.70 mmol/L, total cholesterol ≥ 5.17 mmol/L, low-density lipoprotein cholesterol ≥ 3.37 mmol/L, and/or self-reported hyperlipidemia. Hypertension was defined as blood pressure ≥ 140/90 mmHg, self-reported hypertension, or taking blood pressure medications. To assess the overall diet quality, a healthy diet score was created by including five food groups derived from the touchscreen questionnaire [35]: vegetables (< median: 0, ≥ median: 1), fruits (< median: 0, ≥ median: 1), fish (< median: 0, ≥ median: 1), unprocessed red meats (< median: 1, ≥ median: 0), and processed meats (< median: 1, ≥median: 0). While the touchscreen dietary questionnaire does not allow for calculation for total energy intake, it has been shown to reliably rank participants according to intakes of the main food groups [36].

Body height, weight, waist circumference, and hip circumference were measured following a standard procedure, and body mass index (BMI, kg/m^2^) and waist to hip ratio were calculated. Other adiposity measures including body fat percentage, body fat mass, and body fat-free mass were estimated by impedance measurement.

### Statistical analysis

Baseline characteristics of the study participants overall and across the frequency of adding salt to foods were shown in means ± standard deviations (SDs) for continuous variables and percentages for categorical variables. The associations here were descriptive by nature as the goal was to characterize study participants rather than test a hypothesis, so we did not indicate statistical significance.

The associations of the frequency of adding salt to foods with risk of incident MASLD, cirrhosis, and HCC were computed as hazard ratios (HRs) and 95% confidence intervals (CIs) by Cox proportional hazards models with follow-up time as the timescale. The proportional hazards assumption was confirmed by the likelihood ratio test comparing the models with and without an interaction term between follow-up time and the frequency of adding salt to foods, and no violations were observed. Four multivariable models were fitted. The first model adjusted for age, sex, ethnicity, and Townsend deprivation index. The second model adjusted for smoking status, alcohol drinking status, physical activity, sedentary time, CVD, cancer, diabetes, hyperlipidemia, and hypertension. The third model (primary model) further included healthy diet score. The final model additionally adjusted for BMI, a potential mediator. Missing data were imputed median values for continuous variables and coded as indicator variables for categorical variables. The linear trend was calculated by using the frequency of adding salt to foods (never/rarely: 1, sometimes: 2, usually: 3, and always: 4) as an ordinal variable. To better understand how the risk of outcomes may change over time and if confounding was adequately addressed (risk curves according to different frequencies of adding salt to foods should not split near time 0 before the exposure has had sufficient time to have an effect), we used inverse probability weights for covariate adjustment to create adjusted survival curves [37, 38].

To assess potential effect modifications, we conducted subgroup analyses based on the baseline covariates including age (< 60 or ≥ 60 years), sex (males or females), ethnicity (White or others), Townsend deprivation index (< median or ≥ median), smoking status (current, previous, never), alcohol drinking status (current, previous, never), physical activity (< 600 or ≥ 600 MET-minutes/week), sedentary time (< median or ≥ median), CVD (yes or no), cancer (yes or no), diabetes (yes or no), hyperlipidemia (yes or no), hypertension (yes or no), healthy diet score (< median or ≥ median), and BMI (< 25 or ≥ 25 kg/m^2^). Cross-product terms were created between these stratification factors and the frequency of adding salt to foods, and interactions were evaluated using the likelihood ratio test.

Several sensitivity analyses were conducted to assess the robustness of our findings. First, to minimize potential reverse causality, we excluded the outcomes of interest that occurred within the first two years of follow-up. Second, we excluded participants who had major dietary changes in the last five years based on the question “Have you made any major changes to your diet in the last 5 years?”. Third, to minimize confounding due to alcohol intake, we excluded MASLD patients with significant alcohol intake (weekly intake ≥ 210 g in males or ≥ 140 g in females [39]; *n* = 1,671) and adjusted for alcohol intake in the analyses. Fourth, to more accurately capture diet quality and consider total energy intake, we adjusted for fruits, vegetables, red and processed meats, fish, sugar-sweetened beverages, and total energy intake collected using a validated 24-hour dietary assessment tool [40] instead of the healthy diet score derived from the touchscreen questionnaire. Fifth, we used general linear models to analyze the associations of the frequency of adding salt to foods with sodium and potassium in the urine. Sixth, the association between urinary sodium and outcomes was assessed using multivariable Cox models. Finally, we analyzed the association between adding salt to foods and MASLD defined by PDFF using logistic regression models, adjusted for the same covariates as the above Cox models.

To examine whether adiposity mediates the associations of adding salt to foods with outcomes, we assessed the proportion of the associations mediated by different adiposity measures (including BMI, waist to hip ratio, body fat percentage, body fat mass, and body fat-free mass) using the SAS %MEDIATE macro. In addition, we explored the mediation effects of diabetes and alcohol intake on such associations.

Statistical analyses were performed using SAS 9.4 (SAS Institute Inc., Cary, NC, USA). A 2-sided *P* value of < 0.05 was considered statistically significant.

## Results

### Baseline characteristics

Table 1 presents the baseline characteristics of 492,265 participants (male: 45.3%, mean age: 56.5 ± 8.1 years). Compared with participants who never/rarely added salt to foods, those with a higher frequency of adding salt to foods were more likely to be males, had a higher BMI, were less likely to be White, were more likely to currently smoke, and had higher Townsend deprivation index and sedentary time. They also had a higher prevalence of CVD, cancer, and diabetes, but a lower prevalence of hypertension, and poor diet quality. Supplementary Table 2 shows the baseline cardiometabolic risk factors for incident MASLD cases.

**Table 1:** Associations of the frequency of adding salt to foods with risk of incident MASLD, cirrhosis, and HCC 1

Characteristics	Overall	Frequency of adding salt to foods			
		Never/rarely	Sometimes	Usually	Always
Number of participants	492,265	273,809	138,065	56,976	23,415
Age (years)	56.5 ± 8.1	56.5 ± 8.1	56.4 ± 8.1	57.0 ± 8.0	56.0 ± 8.3
Sex (male, %)	45.3	43.7	45.7	50.7	47.7
BMI (kg/m^2^)	27.4 ± 4.8	27.2 ± 4.7	27.6 ± 4.8	27.8 ± 4.8	28.1 ± 5.1
Ethnicity (white, %)	94.3	95.5	93.4	93.4	87.4
Smoking status (%)					
Current	10.2	7.8	11.0	14.8	22.4
Previous	34.5	32.5	36.0	39.5	36.2
Never	54.9	59.3	52.5	45.4	40.8
Missing	0.38	0.33	0.42	0.42	0.61
Alcohol drinking status (%)					
Current	92.0	91.9	92.7	92.5	87.5
Previous	3.44	3.58	3.04	3.21	4.75
Never	4.48	4.45	4.14	4.19	7.57
Missing	0.11	0.08	0.13	0.14	0.20
Townsend deprivation index	-1.33 ± 3.07	-1.51 ± 2.98	-1.24 ± 3.10	-1.12 ± 3.15	-0.25 ± 3.48
PA (MET-minutes/week)	2651.0 ± 2708.4	2642.0 ± 2650.6	2639.8 ± 2719.2	2643.2 ± 2765.1	2852.6 ± 3164.5
Sedentary time (hours/day)	4.79 ± 2.45	4.65 ± 2.36	4.87 ± 2.46	5.06 ± 2.52	5.41 ± 2.87
Cardiovascular disease (%)	7.90	7.81	7.68	8.29	9.22
Cancer (%)	8.71	8.63	8.70	9.10	8.71
Diabetes (%)	6.06	5.85	6.27	6.22	6.79
Hyperlipidemia (%)	80.3	79.8	80.7	81.7	80.2
Hypertension (%)	55.4	56.0	54.4	55.0	54.3
Healthy diet score	3.00 ± 1.27	3.13 ± 1.25	2.93 ± 1.26	2.73 ± 1.27	2.50 ± 1.26

Abbreviations: BMI, body mass index; MET, metabolic equivalent; PA, physical activity

^1^ Continuous variables were expressed as means ± standard deviations and categorical variables as %

### Association between adding salt to foods and risk of liver-related disorders

**Fig. 1:**
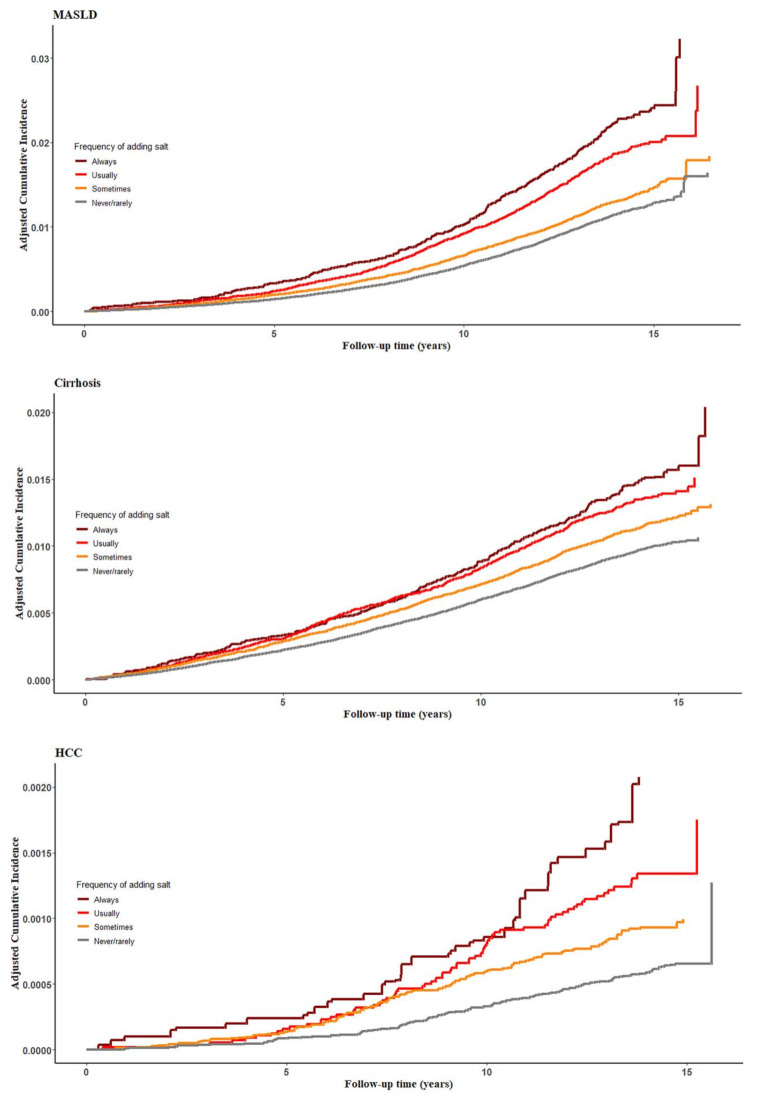
The inverse probability weighted survival curves for the associations of the frequency of adding salt to foods with risk of MASLD, cirrhosis, and HCC. Adjusted for age, sex, ethnicity, Townsend deprivation index, smoking status, alcohol drinking status, physical activity, sedentary time, cardiovascular disease, cancer, diabetes, hyperlipidemia, hypertension, and healthy diet score. Abbreviations: HCC, hepatocellular carcinoma; MASLD, metabolic dysfunction-associated steatotic liver disease

During a median follow-up of 13 years, we documented 7,005 MASLD cases, 5,546 cirrhosis cases, and 413 HCC cases. The covariate-adjusted risks for MASLD, cirrhosis, and HCC across the frequency of adding salt to foods are shown in Table 2. After adjusting for age, sex, ethnicity, and Townsend deprivation index, the HRs (95% CIs) of incident MASLD were 1.00 (reference) for never/rarely, 1.11 (1.05, 1.18) for sometimes, 1.28 (1.19, 1.37) for usually, and 1.58 (1.44, 1.73) for always, with a *P* for trend < 0.0001. Further adjustment for lifestyle factors, disease status, and diet quality made the associations slightly attenuated; the adjusted HRs (95% CIs) were 1.00 (reference) for never/rarely, 1.08 (1.02, 1.14) for sometimes, 1.22 (1.13, 1.31) for usually, and 1.40 (1.27, 1.53) for always. Additional adjustment for BMI attenuated these associations but remained significant; the corresponding HRs (95% CIs) of incident MASLD across the increasing frequency of adding salt to foods were 1.00 (reference), 1.04 (0.99, 1.10), 1.16 (1.08, 1.24), and 1.33 (1.21, 1.46) (*P* for trend < 0.0001). For cirrhosis and HCC, similar association patterns were observed; the fully adjusted HRs (95% CIs) of cirrhosis were 1.00 (reference) for never/rarely, 1.11 (1.04, 1.18) for sometimes, 1.09 (1.00, 1.18) for usually, and 1.32 (1.18, 1.47) for always (*P* for trend < 0.0001); the corresponding HRs (95% CIs) of HCC were 1.00 (reference), 1.26 (1.00, 1.58), 1.45 (1.10, 1.93), and 2.25 (1.60, 3.16), with *P* for trend < 0.0001. The absolute risks (cumulative incidence) of MASLD, cirrhosis, and HCC increased over time, and participants with a higher frequency of adding salt to foods generally had higher risks (Fig. 1).

**Table 2:** Stratified analysis of association of the frequency of adding salt to foods with risk of incident MASLD 1

	Frequency of adding salt to foods				*P* for trend ^2^
	Never/rarely	Sometimes	Usually	Always	
Number of participants	273,809	138,065	56,976	23,415	
**MASLD**					
Number of cases	3,502	2,015	968	520	
Person-years	3,677,875	1,849,840	759,142	309,094	
Incidence per 1000 person-years	0.95	1.09	1.28	1.68	
Model 1	1.00 (reference)	1.11 (1.05, 1.18)	1.28 (1.19, 1.37)	1.58 (1.44, 1.73)	< 0.0001
Model 2	1.00 (reference)	1.09 (1.04, 1.16)	1.24 (1.16, 1.34)	1.44 (1.31, 1.58)	< 0.0001
Model 3	1.00 (reference)	1.08 (1.02, 1.14)	1.22 (1.13, 1.31)	1.40 (1.27, 1.53)	< 0.0001
Model 4	1.00 (reference)	1.04 (0.99, 1.10)	1.16 (1.08, 1.24)	1.33 (1.21, 1.46)	< 0.0001
**Cirrhosis**					
Number of cases	2,801	1654	719	372	
Person-years	3,684,446	1,853,559	761,046	310,186	
Incidence per 1000 person-years	0.76	0.89	0.94	1.20	
Model 1	1.00 (reference)	1.16 (1.09, 1.23)	1.17 (1.08, 1.27)	1.50 (1.34, 1.67)	< 0.0001
Model 2	1.00 (reference)	1.14 (1.07, 1.21)	1.13 (1.04, 1.23)	1.38 (1.24, 1.54)	< 0.0001
Model 3	1.00 (reference)	1.13 (1.06, 1.20)	1.11 (1.02, 1.21)	1.34 (1.20, 1.50)	< 0.0001
Model 4	1.00 (reference)	1.11 (1.04, 1.18)	1.09 (1.00, 1.18)	1.32 (1.18, 1.47)	< 0.0001
**HCC**					
Number of cases	177	124	69	43	
Person-years	3,691,846	1,858,045	762,987	311,180	
Incidence per 1000 person-years	0.05	0.07	0.09	0.14	
Model 1	1.00 (reference)	1.36 (1.08, 1.71)	1.63 (1.23, 2.15)	2.68 (1.91, 3.74)	< 0.0001
Model 2	1.00 (reference)	1.32 (1.05, 1.67)	1.58 (1.20, 2.10)	2.50 (1.78, 3.51)	< 0.0001
Model 3	1.00 (reference)	1.30 (1.03, 1.63)	1.52 (1.14, 2.01)	2.34 (1.66, 3.30)	< 0.0001
Model 4	1.00 (reference)	1.26 (1.00, 1.58)	1.45 (1.10, 1.93)	2.25 (1.60, 3.16)	< 0.0001

Abbreviations: HCC, hepatocellular carcinoma; MASLD, metabolic dysfunction-associated steatotic liver disease

^1^ Values are hazard ratios (95% confidence interval) unless otherwise indicated, calculated by Cox proportional hazards models

^2^*P* for trend was calculated by using the frequency of adding salt to foods (never/rarely: 1, sometimes: 2, usually: 3, and always: 4) as an ordinal variable

Model 1 was adjusted for age, sex, ethnicity, and Townsend deprivation index

Model 2 was further adjusted for smoking status, alcohol drinking status, physical activity, sedentary time, cardiovascular disease, cancer, diabetes, hyperlipidemia, and hypertension

Model 3 was additionally adjusted for healthy diet score

Model 4 was additionally adjusted for body mass index

### Subgroup analyses

**Fig. 2:**
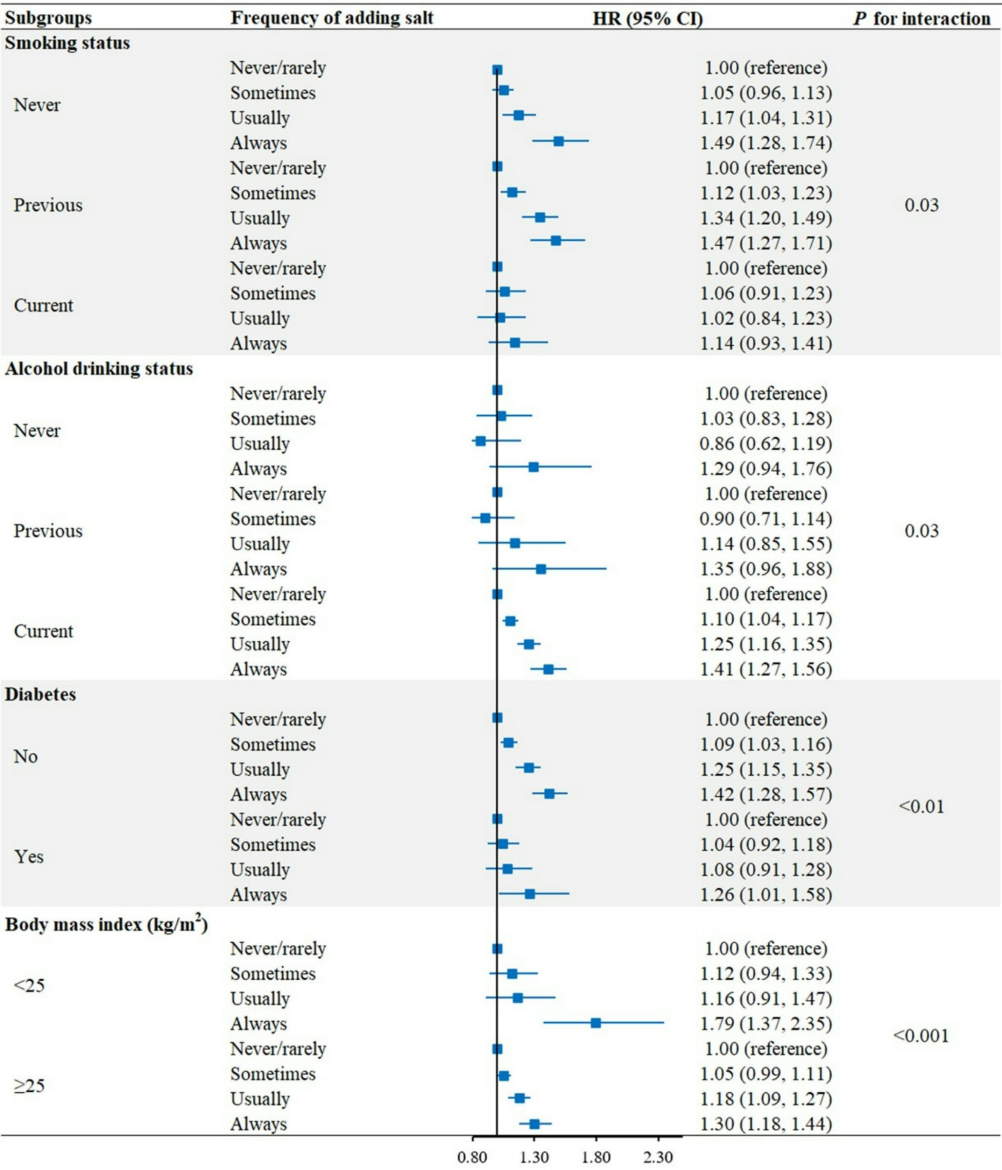
Associations of the frequency of adding salt to foods and smoking, alcohol drinking, diabetes, as well as body mass index with risk of metabolic dysfunction-associated steatotic liver disease. Cox proportional hazards models adjusted for age, sex, ethnicity, Townsend deprivation index, smoking status, alcohol drinking status, physical activity, sedentary time, cardiovascular diseases, cancer, diabetes, hyperlipidemia, hypertension, and healthy diet score. *P* for interaction was calculated by adding interaction terms to the Cox models

Table 3 presents the stratified analysis of the association between the frequency of adding salt to foods and risk of incident MASLD. No significant interactions were observed between the frequency of adding salt to foods and age, sex, ethnicity, Townsend deprivation index, physical activity, sedentary time, CVD, cancer, hyperlipidemia, hypertension, and healthy diet score in relation to risk of MASLD. In contrast, non-current smokers, current alcohol drinkers, and those with non-diabetes or lower BMI showed stronger associations of the frequency of adding salt to foods with the risk of MASLD (Fig. 2, P for interaction < 0.05). After excluding MASLD cases with significant alcohol intake, a stronger association remained in current alcohol drinkers than in previous or never drinkers (Table 3). Furthermore, associations between the frequency of adding salt to foods and cirrhosis/HCC were generally consistent across different subgroups, except for age on cirrhosis where the positive association was stronger in young participants (Supplementary Table 3) and sex on HCC where the positive association was observed only in males (Supplementary Table 4).

**Table 3:** Baseline characteristics of the study participants overall and according to the frequency of adding salt to foods 1

Subgroup	Frequency of adding salt to foods				*P* for trend ^2^	*P* for interaction ^3^
	Never/rarely	Sometimes	Usually	Always		
**Age** (years)						
<60 (*n* = 278,848)	1.00 (reference)	1.08 (1.01, 1.17)	1.29 (1.17, 1.42)	1.37 (1.21, 1.55)	< 0.0001	0.46
≥60 (*n* = 213,417)	1.00 (reference)	1.08 (0.99, 1.17)	1.13 (1.02, 1.26)	1.43 (1.24, 1.66)	< 0.0001	
**Sex**						
Male (*n* = 22,2870)	1.00 (reference)	1.05 (0.97, 1.14)	1.21 (1.10, 1.34)	1.41 (1.23, 1.62)	< 0.0001	0.44
Female (*n* = 26,9395)	1.00 (reference)	1.11 (1.03, 1.19)	1.22 (1.10, 1.35)	1.37 (1.20, 1.56)	< 0.0001	
**Ethnicity**						
White (*n* = 464,104)	1.00 (reference)	1.07 (1.01, 1.14)	1.23 (1.14, 1.32)	1.42 (1.28, 1.57)	< 0.0001	0.13
Others (*n* = 28,161)	1.00 (reference)	1.22 (0.99, 1.49)	1.09 (0.82, 1.45)	1.29 (0.96, 1.73)	0.10	
**Townsend deprivation index**						
< Median (*n* = 245,760)	1.00 (reference)	1.12 (1.03, 1.23)	1.20 (1.07, 1.36)	1.55 (1.32, 1.83)	< 0.0001	0.28
≥Median (*n* = 246,505)	1.00 (reference)	1.05 (0.98, 1.13)	1.22 (1.11, 1.33)	1.33 (1.18, 1.49)	< 0.0001	
**Smoking status**						
Current (*n* = 50,316)	1.00 (reference)	1.06 (0.91, 1.23)	1.02 (0.84, 1.23)	1.14 (0.93, 1.41)	0.30	0.03
Previous (*n* = 169,705)	1.00 (reference)	1.12 (1.03, 1.23)	1.34 (1.20, 1.49)	1.47 (1.27, 1.71)	< 0.0001	
Never (*n* = 270,375)	1.00 (reference)	1.05 (0.96, 1.13)	1.17 (1.04, 1.31)	1.49 (1.28, 1.74)	< 0.0001	
**Alcohol drinking status**						
Current (*n* = 452,706)	1.00 (reference)	1.10 (1.04, 1.17)	1.25 (1.16, 1.35)	1.41 (1.27, 1.56)	< 0.0001	0.03
Previous (*n* = 16,955)	1.00 (reference)	0.90 (0.71, 1.14)	1.14 (0.85, 1.55)	1.35 (0.96, 1.88)	0.13	
Never (*n* = 22,073)	1.00 (reference)	1.03 (0.83, 1.28)	0.86 (0.62, 1.19)	1.29 (0.94, 1.76)	0.43	
**Alcohol drinking status** ^4^						
Current (*n* = 451,035)	1.00 (reference)	1.09 (1.01, 1.16)	1.23 (1.12, 1.35)	1.37 (1.20, 1.55)	< 0.0001	0.02
Previous (*n* = 16,955)	1.00 (reference)	0.90 (0.71, 1.14)	1.14 (0.85, 1.55)	1.35 (0.96, 1.88)	0.13	
Never (*n* = 22,073)	1.00 (reference)	1.03 (0.83, 1.28)	0.86 (0.62, 1.19)	1.29 (0.94, 1.76)	0.43	
**Physical activity** (MET-minutes/week)						
<600 (*n* = 73,905)	1.00 (reference)	1.03 (0.91, 1.17)	1.17 (0.99, 1.37)	1.32 (1.07, 1.62)	< 0.01	0.14
≥600 (*n* = 418,360)	1.00 (reference)	1.09 (1.03, 1.16)	1.23 (1.13, 1.33)	1.41 (1.27, 1.57)	< 0.0001	
**Sedentary time** (hours/day)						
< Median (*n* = 235,418)	1.00 (reference)	1.09 (1.00, 1.19)	1.23 (1.09, 1.39)	1.46 (1.24, 1.72)	< 0.001	0.18
≥Median (*n* = 256,847)	1.00 (reference)	1.08 (1.01, 1.16)	1.21 (1.11, 1.32)	1.39 (1.24, 1.56)	< 0.0001	
**Cardiovascular disease**						
Yes (*n* = 38,877)	1.00 (reference)	0.96 (0.83, 1.11)	1.14 (0.94, 1.37)	1.35 (1.08, 1.70)	0.02	0.19
No (*n* = 453,388)	1.00 (reference)	1.10 (1.04, 1.17)	1.23 (1.14, 1.33)	1.41 (1.27, 1.56)	< 0.0001	
**Cancer**						
Yes (*n* = 42,863)	1.00 (reference)	1.07 (0.90, 1.27)	1.32 (1.06, 1.64)	1.19 (0.86, 1.64)	0.03	0.78
No (*n* = 449,402)	1.00 (reference)	1.08 (1.02, 1.15)	1.20 (1.12, 1.30)	1.42 (1.29, 1.57)	< 0.0001	
**Diabetes**						
Yes (*n* = 29,809)	1.00 (reference)	1.04 (0.92, 1.18)	1.08 (0.91, 1.28)	1.26 (1.01, 1.58)	0.050	< 0.01
No (*n* = 46,2456)	1.00 (reference)	1.09 (1.03, 1.16)	1.25 (1.15, 1.35)	1.42 (1.28, 1.57)	< 0.0001	
**Hyperlipidemia**						
Yes (*n* = 395,421)	1.00 (reference)	1.08 (1.02, 1.15)	1.19 (1.10, 1.28)	1.38 (1.24, 1.53)	< 0.0001	0.12
No (*n* = 96,844)	1.00 (reference)	1.09 (0.94, 1.26)	1.40 (1.16, 1.68)	1.49 (1.17, 1.90)	< 0.001	
**Hypertension**						
Yes (*n* = 272,577)	1.00 (reference)	1.09 (1.02, 1.16)	1.22 (1.12, 1.33)	1.45 (1.29, 1.62)	< 0.0001	0.96
No (*n* = 219,688)	1.00 (reference)	1.07 (0.96, 1.18)	1.20 (1.06, 1.37)	1.28 (1.07, 1.52)	< 0.001	
**Healthy diet score**						
< Median (*n* = 159,058)	1.00 (reference)	1.02 (0.93, 1.12)	1.17 (1.05, 1.31)	1.35 (1.18, 1.55)	< 0.0001	0.41
≥Median (*n* = 333,207)	1.00 (reference)	1.12 (1.04, 1.20)	1.24 (1.13, 1.36)	1.44 (1.26, 1.64)	< 0.0001	
**Body mass index** (kg/m^2^)						
<25 (*n* = 162,107)	1.00 (reference)	1.12 (0.94, 1.33)	1.16 (0.91, 1.47)	1.79 (1.37, 2.35)	< 0.001	< 0.001
≥25 (*n* = 330,158)	1.00 (reference)	1.05 (0.99, 1.11)	1.18 (1.09, 1.27)	1.30 (1.18, 1.44)	< 0.0001	

Abbreviations: MASLD, metabolic dysfunction-associated steatotic liver disease; MET, metabolic equivalent

^1^ Values are hazard ratios (95% confidence interval) unless otherwise indicated, calculated by Cox proportional hazards models

^2^*P* for trend was calculated by using the frequency of adding salt to foods (never/rarely: 1, sometimes: 2, usually: 3, and always: 4) as an ordinal variable

^3^*P* for interaction was calculated by adding interaction terms to the Cox models

^4^ Excluded MASLD cases with significant alcohol intake (weekly intake ≥ 210 g in males or ≥ 140 g in females)

Adjusted for age, sex, ethnicity, Townsend deprivation index, smoking status, alcohol drinking status, physical activity, sedentary time, cardiovascular disease, cancer, diabetes, hyperlipidemia, hypertension, and healthy diet score

### Sensitivity analyses

The results did not appreciably change in a series of sensitivity analyses including the exclusion of incident events that occurred within the first two years of follow-up (Supplementary Table 5), the exclusion of participants who had major dietary changes in the last five years (Supplementary Table 6), the exclusion of MASLD cases with excessive alcohol intake and adjustment for alcohol intake (Supplementary Table 7), or the adjustment for dietary intake assessed by 24-hour dietary assessment (Supplementary Table 8). In addition, spot urinary sodium concentration and estimated 24-hour sodium excretion amount increased with increased frequencies of adding salt to foods (Supplementary Table 9). Moreover, significant positive associations were observed between spot urinary sodium and estimated 24-hour sodium excretion and risk of MASLD, U-shape associations were seen for cirrhosis, while no associations were found for HCC (Supplementary Table 10). The association between adding salt to foods and PDFF-defined MASLD was similar to that of MASLD identified by ICD-10 codes (Supplementary Table 11).

### Mediation analyses

Overall, the positive associations between the frequency of adding salt to foods with the risk of incident MASLD, cirrhosis, and HCC were partly mediated by adiposity (Table 4). For MASLD, more than 20% were explained by BMI (22.1%; 95% CI: 16.1%, 29.5%), waist to hip ratio (23.0%; 95% CI: 16.9%, 30.4%), body fat percentage (28.2%; 95% CI: 21.0%, 36.7%), and body fat mass (21.6%; 95% CI: 15.8%, 28.8%), while body fat-free mass only mediated 2.4% (95% CI: 1.1%, 5.3%). For cirrhosis and HCC, the mediated effects were derived by BMI, waist to hip ratio, body fat percentage, and body fat mass, rather than body fat-free mass. Furthermore, no mediation effects were observed for diabetes in the associations, while alcohol intake mediated the associations of the frequency of adding salt to foods with risks of cirrhosis and HCC, with proportions of 19.5% (11.8%, 30.5%) and 8.2% (4.5%, 14.4%) (Supplementary Table 12).

**Table 4:** Mediation analyses of adiposity measures on the associations of the frequency of adding salt to foods with the risk of incident MASLD, cirrhosis, and HCC

	Hazard ratio (95% confidence interval)		Proportion mediated	*P* value	
	Unadjusted for the factor	Adjusted for the factor			
**MASLD**					
Body mass index	1.11 (1.08, 1.14)	1.08 (1.05, 1.11)	22.1% (16.1%, 29.5%)	< 0.0001	
Waist to hip ratio	1.11 (1.08, 1.14)	1.08 (1.05, 1.11)	23.0% (16.9%, 30.4%)	< 0.0001	
Body fat percentage	1.11 (1.08, 1.14)	1.07 (1.05, 1.10)	28.2% (21.0%, 36.7%)	< 0.0001	
Body fat mass	1.11 (1.08, 1.14)	1.08 (1.05, 1.11)	21.6% (15.8%, 28.8%)	< 0.0001	
Body fat-free mass	1.11 (1.08, 1.14)	1.10 (1.07, 1.13)	2.4% (1.1%, 5.3%)	< 0.01	
**Cirrhosis**					
Body mass index	1.08 (1.05, 1.12)	1.07 (1.04, 1.11)	10.4% (6.7%, 15.8%)	< 0.0001	
Waist to hip ratio	1.08 (1.05, 1.12)	1.07 (1.04, 1.10)	16.2% (10.7%, 23.8%)	< 0.0001	
Body fat percentage	1.08 (1.05, 1.12)	1.07 (1.04, 1.11)	9.7% (6.2%, 15.1%)	< 0.0001	
Body fat mass	1.08 (1.05, 1.12)	1.07 (1.04, 1.11)	10.4% (6.7%, 15.8%)	< 0.0001	
Body fat-free mass	1.08 (1.05, 1.12)	1.08 (1.05, 1.11)	None	-	
**HCC**					
Body mass index	1.30 (1.17, 1.44)	1.27 (1.15, 1.41)	6.6% (3.9%, 11.0%)	< 0.0001	
Waist to hip ratio	1.30 (1.17, 1.44)	1.27 (1.15, 1.41)	6.6% (3.9%, 11.0%)	< 0.0001	
Body fat percentage	1.30 (1.17, 1.44)	1.26 (1.14, 1.40)	9.7% (5.8%, 15.6%)	< 0.0001	
Body fat mass	1.30 (1.17, 1.44)	1.27 (1.15, 1.41)	7.6% (4.6%, 12.3%)	< 0.0001	
Body fat-free mass	1.30 (1.17, 1.44)	1.29 (1.17, 1.44)	None	-	

Abbreviations: HCC, hepatocellular carcinoma; MASLD, metabolic dysfunction-associated steatotic liver disease

All Cox models were adjusted for age, sex, ethnicity, Townsend deprivation index, smoking status, alcohol drinking status, physical activity, sedentary time, cardiovascular disease, cancer, diabetes, hyperlipidemia, hypertension, and healthy diet score

Because the transform the macro uses to get the confidence interval for the proportion mediated does not go below zero, the confidence interval does not contain the null value (0) but a non-significant *P* value. Pay attention to the *P* value, not the confidence interval

## Discussion

In this large prospective cohort of the UK Biobank, we found strong positive associations of the frequency of adding salt to foods with risks of MASLD, cirrhosis, and HCC, consistent across multiple sensitivity analyses. The associations with MASLD appeared to be stronger in participants who were non-current smokers, current alcohol drinkers, did not have prevalent diabetes, and had lower BMI, compared with their counterparts. In addition, our results showed that the positive associations were partly mediated by adiposity measures including BMI, waist to hip ratio, body fat percentage, body fat mass, and body fat-free mass (only 2.4% for MASLD).

To the best of our knowledge, this is the first study that investigates the association between the frequency of adding salt to foods and risk of liver-related disorders. Several previous studies have found that the frequency of adding salt to foods was associated with increased risks of premature mortality [17], CVD [18, 23, 24], type 2 diabetes [20, 22], chronic kidney disease [41], gastric cancer [42], and sleep apnea [43]. This study presents new findings on the frequency of adding salt to foods in relation to MASLD, cirrhosis, and HCC, which is an important extension of previous studies. Our findings are supported by previous cross-sectional studies [8–14], in which positive associations of sodium intake measured by dietary survey or urine samples with MASLD were observed. Another case-control study conducted in France indicated that dietary sodium intake had a significant positive association with HCC in cirrhotic patients [44]. In addition, prospective cohort studies conducted in China showed that higher perceived salt intake (assessed by asking participants to rate their habitual daily salt intake as low, medium, or high) was positively associated with risks of MASLD [45] and primary liver cancer [46]. However, results from the Health Professionals Follow-up Study and the Nurses’ Health Study indicated that dietary sodium intake did not have an association with the risk of HCC [47]. In contrast, no previous study focused on the association between sodium intake and risk of cirrhosis. Thus, future studies are encouraged to confirm our findings. Because adding salt to foods is a common eating behavior and can reflect an individual’s long-term preference for salty-tasting foods and habitual salt intake (97-99% sodium chloride) [19], our study provides novel evidence for the link between sodium intake and liver-related disorders. Furthermore, an important public health nutrition message from this study is that liver-related disorders may be prevented by low sodium intake. Because daily salt intake is strongly associated with salt-intake-related behavior, public health efforts should begin with improving salt literacy and awareness [48].

Interestingly, we observed a stronger positive association between the frequency of adding salt to foods and the risk of MASLD in participants who were non-current smokers, had non-diabetes, or had lower BMI. This phenomenon might be because participants who were current smokers, with diabetes, as well as overweight and/or obese might have had a high metabolic impairment characterized by oxidative stress, chronic inflammation, and insulin resistance. Thus, the adverse health effects of adding salt to foods on MASLD can be overshadowed by metabolic impairment-associated oxidative stress and systemic chronic inflammation among current smokers, individuals with diabetes, or those with higher BMI. Because current smokers, individuals with prevalent diabetes, or those with higher BMI had a higher risk of developing MASLD, the relatively weaker risk might be translated into a substantial reduction in the absolute rate in this high-risk group of participants. Furthermore, the positive association was stronger in current alcohol drinkers than in previous and never drinkers. A previous study showed that alcohol consumers were more likely to consume salt than alcohol abstainers [49]. In addition, current alcohol drinking was positively associated with risk of MASLD [50]. This collectively suggests that salt intake has an additive effect with alcohol intake, increasing the risk of MASLD. Therefore, our finding highlights the importance of reducing salt intake especially in current alcohol drinkers. Furthermore, we observed that the significant positive association between salt added to foods and HCC exists only in males. This may be due to the relatively low number of HCC cases in females in the current study, which reduces the statistical power to detect the association. Also, the association between salt added to foods and cirrhosis was stronger in younger participants compared to older participants.

Mechanistically, the potential detrimental effects of adding salt to foods on MASLD and its liver-related complications may involve several explanations. First, an experiment with mice indicated that high salt intake can activate the aldose reductase pathway in the liver and cause leptin resistance and MASLD by stimulating endogenous fructose production and metabolism [51]. Second, high salt intake can cause the accumulation of liver fat by the dysfunction of the renin-angiotensin-aldosterone system [52]. Third, a high sodium intake reduces the expression of sirtuin 3, thereby worsening the development of MASLD [53]. Taken together, salt-induced MASLD progression can contribute to its complications including cirrhosis and HCC.

As expected, the associations of the frequency of adding salt to foods with the risk of incident MASLD, cirrhosis, and HCC were partly mediated by adiposity. This is supported by a cross-sectional study in Korea, showing that the positive association between dietary sodium intake measured by food frequency questionnaire and MASLD was partly mediated by BMI and body fat percentage [11]. In contrast, other adiposity measures including waist to hip ratio, body fat mass, and body fat-free mass were not considered in that study. In the current study, we showed that the adiposity mediation effect was mainly due to body fat mass rather than body fat-free mass, consistent with our previous study on diabetes [20]. Furthermore, exploratory analyses suggested that adding salt to foods contributed to cirrhosis and HCC partly through its association with alcohol intake, underscoring the critical role of alcohol reduction in preventing these conditions.

The strengths of this study included its large sample size, long follow-up time, low dropout rate, and a unique method to assess habitual sodium intake compared with previous studies. In addition, we used inverse probability weights to plot adjusted survival curves [37], which provide information about the absolute risks [38] and help understand the population-level impact of the exposure on the outcomes.

This study had several limitations. First, information on the frequency of adding salt to foods was collected once at baseline so individuals’ behaviors may have changed over time, which usually leads to an underestimation of the associations. Second, adding salt to foods at the table may be a marker of an unhealthy diet and lifestyle. However, adding salt to foods is a promising indicator of habitual sodium intake in relation to human health [19]. Moreover, our results are robust in multivariable models and subgroup analyses by lifestyle factors. Third, data on the exposure and some covariates were from the self-reported questionnaire and could be subject to recall bias and measurement errors. Fourth, MASLD was defined by hospital admissions and death records, and thus UK Biobank analysis primarily included severe cases of MASLD [28, 54]. However, similar results were yielded when defining MASLD using liver PDFF, a quantitative biomarker to assess liver fat content [25, 26]. Fifth, we did not apply the new nomenclature and diagnostic criteria for MASLD to this study cohort as the cardiometabolic criteria requested for MASLD definition were only available at baseline. However, MASLD definitions with and without cardiometabolic risk factors can be used interchangeably as > 96% of individuals with non-alcoholic fatty liver disease meet MASLD criteria [1]. Sixth, the study was observational and causality cannot be inferred. Seventh, diabetes might be underreported due to lack of data on fasting glucose measurements. Finally, the UK Biobank participants are predominantly of European ancestry and are individuals who are more health conscious [55], which potentially limits the generalizability of the results to other populations.

## Conclusions

In conclusion, a higher frequency of adding salt to foods was associated with higher risks of incident MASLD, cirrhosis, and HCC. These findings suggest that reducing salt/sodium intake could be a promising strategy for preventing liver-related disorders.

## Electronic supplementary material

Below is the link to the electronic supplementary material.

Supplementary Material 1

## Data Availability

The data that support the findings of this study are available on application to the UK Biobank (www.ukbiobank.ac.uk).

## Abbreviations

BMI: Body mass index
CI: Confidence interval
CVD: Cardiovascular disease
HCC: Hepatocellular carcinoma
HR: Hazard ratio
ICD-10: International Classification of Disease, Tenth Revision
MASLD: Metabolic dysfunction-associated steatotic liver disease
MET: Metabolic equivalent task
PDFF: Proton density fat fraction
SD: Standard deviation
STROBE: Strengthening the Reporting of Observational Studies in Epidemiology

## Code availability

The SAS programs will be made available upon request, pending application and approval.
