# Supplementary material for: Adding salt to foods and risk of metabolic dysfunction-associated steatotic liver disease and other chronic liver diseases
Source: Eur J Nutr. 2025 Jun 21;64(5):224. doi: 10.1007/s00394-025-03745-3 (PMC12182457; doi:10.1007/s00394-025-03745-3)
Supplement: Supplementary file 1 — Supplementary Material 1 [file 394_2025_3745_MOESM1_ESM.docx]

**Online Supplementary Material**

**Adding salt to foods and risk of metabolic dysfunction-associated steatotic liver disease and other chronic liver diseases**

Author Names: Shunming Zhang^*^, Zhenyu Huo, Yan Borné, Emily Sonestedt, Lu Qi^*^

**^*^Correspondence to:** Shunming Zhang, shunming.zhang@med.lu.se; Lu Qi, lqi1@tulane.edu

**Supplementary Figure 1.** Flowchart for selection of study participants from the UK Biobank. Abbreviation: PDFF, proton density fat fraction.


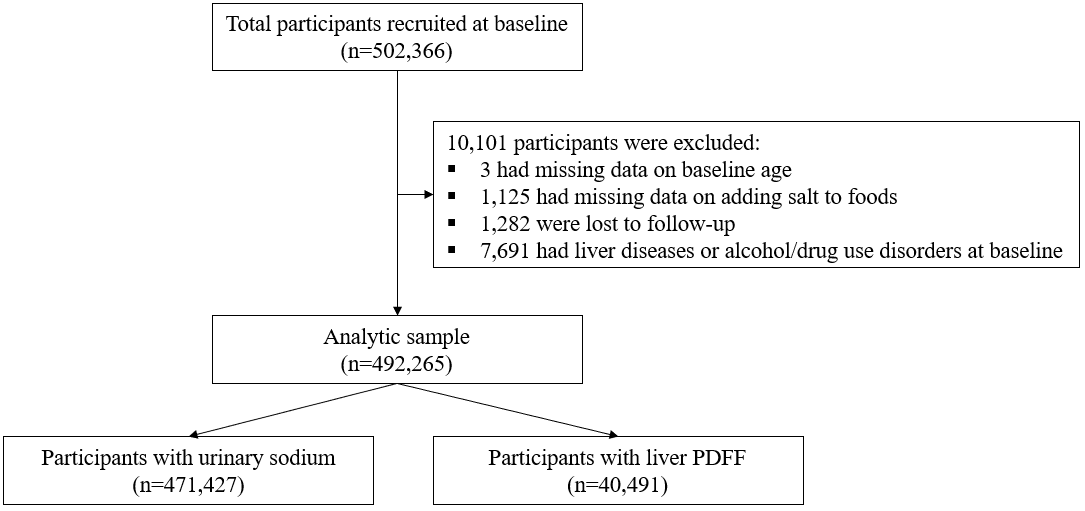


**Supplementary Figure 2.** Directed acyclic graph derived from literature and expert knowledge. The nodes represent specific variables and the arrows denote causal associations between them. The exposure is the frequency of adding salt to foods and the outcomes include metabolic dysfunction-associated steatotic liver disease, cirrhosis, and hepatocellular carcinoma. Personal history of diseases includes cardiovascular disease, cancer, diabetes, hyperlipidemia, and hypertension.


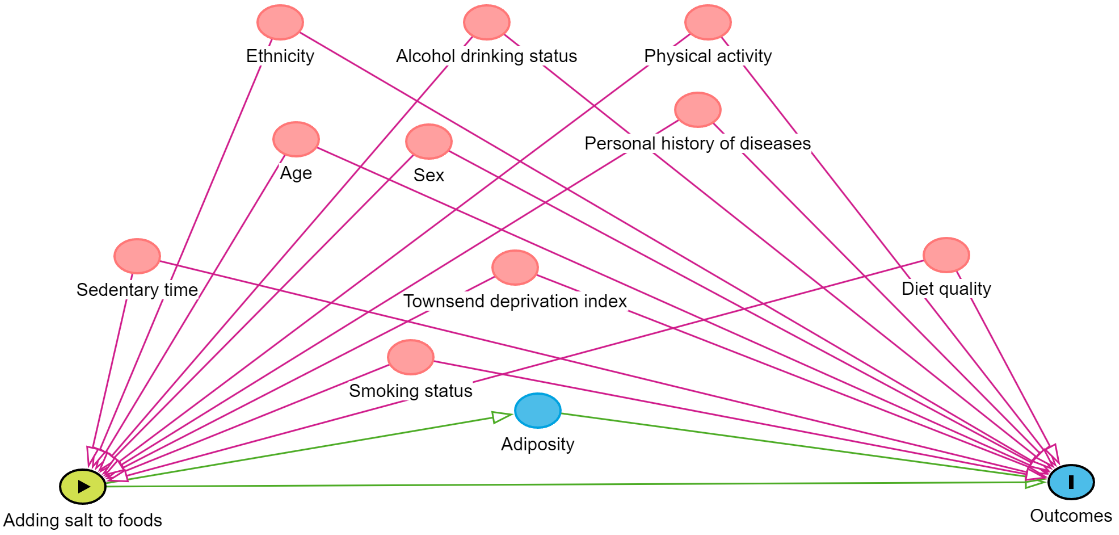


| **Supplementary Table 1.** List of International Classification of Disease codes for liver disease diagnoses | |
| --- | --- |
| Diseases | International Classification of Disease codes |
| Metabolic dysfunction-associated steatotic liver disease | K76.0, K75.8 |
| Cirrhosis | K74.0, K74.1, K74.2, K74.6, K76.6, K76.7, R18, I85.0, I85.9, I86.4, I98.2, I98.3 |
| Hepatocellular carcinoma | C22.0 |
| Liver failure | K72.1, K72.9, K72.0 |
| Alcoholic liver disease (alcohol-related liver disease) | K70 |
| Viral hepatitis | B15, B16, B17, B18, B19 |
| Autoimmune liver disease (autoimmune hepatitis, primary biliary cholangitis, primary sclerosing cholangitis) | K83.0, K74.3, K75.4 |
| Hemochromatosis | E83.1 |
| Wilson | E83.0 |
| Alpha-1-antitrypsin deficiency | E88.0 |
| Budd-Chiari | I82.0, K76.5 |
| Chronic hepatitis, unspecified | K73.9, K73.2 |
| Secondary or unspecified biliary cirrhosis | K74.4, K74.5 |
| Nonspecific reactive hepatitis | K75.2 |
| Toxic liver disease | K71 |
| Codes associated with alcohol use disorder | F10 |
| Codes associated with somatic consequences of alcohol (except alcohol-related liver disease) | E24.4, G62.1, I42.6, K29.2, G31.2, G72.1, K85.2, K86.0, T51.0, T51.9, Y57.3, X65, Z50.2, Z71.4, Z72.1 |
| Codes associated with drug use disorders except nicotine/caffeine | F11, F12, F13, F14, F16, F18, F19 |
| Liver transplantation | Z94.4 |
| References: Hepatology. 2021;74(1):474-482; J Cachexia Sarcopenia Muscle. 2024;15(4):1491-1500. | |

| **Supplementary Table 2.** Baseline characteristics of metabolic dysfunction-associated steatotic liver disease cases ^1^ | |
| --- | --- |
| Characteristics | Metabolic dysfunction-associated steatotic liver disease cases |
| Number of participants | 7,005 |
| BMI (kg/m^2^) | 31.3±5.7 |
| Waist circumference (cm) | 100.5±13.9 |
| Glycated hemoglobin (mmol/mol) | 39.5±10.4 |
| Systolic blood pressure (mmHg) | 140.6±18.0 |
| Diastolic blood pressure (mmHg) | 84.4±10.2 |
| Hypertension (%) | 70.8 |
| Diabetes (%) | 19.2 |
| Hyperlipidemia (%) | 85.4 |
| ^1^ Continuous variables were expressed as means±standard deviations and categorical variables as %. | |

| **Supplementary Table 3.** Stratified analysis of associations of the frequency of adding salt to foods with risk of incident cirrhosis ^1^ | | | | | | |
| --- | --- | --- | --- | --- | --- | --- |
| Subgroup | Frequency of adding salt to foods | | | | *P* for trend ^2^ | *P* for interaction ^3^ |
|  | Never/rarely | Sometimes | Usually | Always |  |  |
| **Age** (years) |  |  |  |  |  |  |
| <60 (n=278,848) | 1.00 (reference) | 1.13 (1.03, 1.25) | 1.23 (1.08, 1.39) | 1.42 (1.20, 1.67) | <0.0001 | 0.04 |
| ≥60 (n=213,417) | 1.00 (reference) | 1.12 (1.03, 1.21) | 1.03 (0.93, 1.15) | 1.27 (1.10, 1.48) | <0.01 |  |
| **Sex** |  |  |  |  |  |  |
| Male (n=22,2870) | 1.00 (reference) | 1.14 (1.05, 1.24) | 1.10 (0.98, 1.23) | 1.42 (1.23, 1.65) | <0.0001 | 0.32 |
| Female (n=26,9395) | 1.00 (reference) | 1.11 (1.02, 1.21) | 1.13 (1.00, 1.28) | 1.23 (1.04, 1.46) | <0.01 |  |
| **Ethnicity** |  |  |  |  |  |  |
| White (n=464,104) | 1.00 (reference) | 1.12 (1.05, 1.19) | 1.12 (1.03, 1.22) | 1.33 (1.19, 1.49) | <0.0001 | 0.53 |
| Others (n=28,161) | 1.00 (reference) | 1.17 (0.90, 1.52) | 0.96 (0.65, 1.41) | 1.34 (0.92, 1.95) | 0.26 |  |
| **Townsend deprivation index** |  |  |  |  |  |  |
| <Median (n=245,760) | 1.00 (reference) | 1.18 (1.08, 1.30) | 1.05 (0.92, 1.20) | 1.38 (1.14, 1.67) | <0.001 | 0.58 |
| ≥Median (n=246,505) | 1.00 (reference) | 1.08 (1.00, 1.17) | 1.15 (1.03, 1.28) | 1.31 (1.14, 1.50) | <0.0001 |  |
| **Smoking status** |  |  |  |  |  |  |
| Current (n=50,316) | 1.00 (reference) | 1.24 (1.05, 1.46) | 1.11 (0.90, 1.36) | 1.36 (1.08, 1.71) | 0.02 | 0.51 |
| Previous (n=169,705) | 1.00 (reference) | 1.06 (0.96, 1.17) | 1.09 (0.96, 1.24) | 1.42 (1.20, 1.68) | <0.001 |  |
| Never (n=270,375) | 1.00 (reference) | 1.15 (1.05, 1.26) | 1.13 (0.99, 1.29) | 1.21 (0.99, 1.47) | <0.01 |  |
| **Alcohol drinking status** |  |  |  |  |  |  |
| Current (n=452,706) | 1.00 (reference) | 1.14 (1.07, 1.22) | 1.13 (1.03, 1.23) | 1.37 (1.21, 1.54) | <0.0001 | 0.25 |
| Previous (n=16,955) | 1.00 (reference) | 0.95 (0.71, 1.26) | 0.86 (0.57, 1.30) | 0.98 (0.60, 1.59) | 0.61 |  |
| Never (n=22,073) | 1.00 (reference) | 1.06 (0.80, 1.40) | 1.04 (0.70, 1.54) | 1.33 (0.87, 2.03) | 0.27 |  |
| **Physical activity** (MET-minutes/week) |  |  |  |  |  |  |
| <600 (n=73,905) | 1.00 (reference) | 1.10 (0.95, 1.27) | 1.03 (0.84, 1.25) | 1.36 (1.07, 1.74) | 0.04 | 0.50 |
| ≥600 (n=418,360) | 1.00 (reference) | 1.13 (1.06, 1.21) | 1.13 (1.03, 1.23) | 1.33 (1.17, 1.50) | <0.0001 |  |
| **Sedentary time** (hours/day) |  |  |  |  |  |  |
| <Median (n=235,418) | 1.00 (reference) | 1.08 (0.98, 1.18) | 1.01 (0.88, 1.16) | 1.26 (1.05, 1.53) | 0.050 | 0.14 |
| ≥Median (n=256,847) | 1.00 (reference) | 1.17 (1.08, 1.26) | 1.18 (1.06, 1.31) | 1.41 (1.23, 1.61) | <0.0001 |  |
| **Cardiovascular disease** |  |  |  |  |  |  |
| Yes (n=38,877) | 1.00 (reference) | 1.03 (0.88, 1.20) | 1.10 (0.89, 1.35) | 1.31 (1.01, 1.70) | 0.06 | 0.60 |
| No (n=453,388) | 1.00 (reference) | 1.15 (1.07, 1.22) | 1.11 (1.02, 1.22) | 1.35 (1.19, 1.52) | <0.0001 |  |
| **Cancer** |  |  |  |  |  |  |
| Yes (n=42,863) | 1.00 (reference) | 1.27 (1.08, 1.49) | 1.36 (1.10, 1.68) | 1.05 (0.74, 1.49) | 0.01 | 0.66 |
| No (n=449,402) | 1.00 (reference) | 1.10 (1.03, 1.18) | 1.07 (0.98, 1.17) | 1.38 (1.23, 1.55) | <0.0001 |  |
| **Diabetes** |  |  |  |  |  |  |
| Yes (n=29,809) | 1.00 (reference) | 1.22 (1.05, 1.42) | 1.27 (1.04, 1.55) | 1.41 (1.08, 1.85) | <0.001 | 0.58 |
| No (n=46,2456) | 1.00 (reference) | 1.11 (1.04, 1.19) | 1.08 (0.99, 1.18) | 1.32 (1.17, 1.49) | <0.0001 |  |
| **Hyperlipidemia** |  |  |  |  |  |  |
| Yes (n=395,421) | 1.00 (reference) | 1.11 (1.04, 1.19) | 1.10 (1.01, 1.21) | 1.39 (1.23, 1.57) | <0.0001 | 0.57 |
| No (n=96,844) | 1.00 (reference) | 1.20 (1.04, 1.38) | 1.15 (0.94, 1.40) | 1.10 (0.83, 1.46) | 0.0818 |  |
| **Hypertension** |  |  |  |  |  |  |
| Yes (n=272,577) | 1.00 (reference) | 1.13 (1.05, 1.22) | 1.13 (1.03, 1.25) | 1.40 (1.23, 1.60) | <0.0001 | 0.17 |
| No (n=219,688) | 1.00 (reference) | 1.11 (1.00, 1.24) | 1.06 (0.91, 1.23) | 1.20 (0.98, 1.47) | 0.0549 |  |
| **Healthy diet score** |  |  |  |  |  |  |
| <Median (n=159,058) | 1.00 (reference) | 1.15 (1.04, 1.28) | 1.12 (0.98, 1.27) | 1.40 (1.19, 1.64) | <0.0001 | 0.43 |
| ≥Median (n=333,207) | 1.00 (reference) | 1.11 (1.03, 1.20) | 1.11 (0.99, 1.23) | 1.28 (1.10, 1.50) | <0.001 |  |
| **Body mass index** (kg/m^2^) |  |  |  |  |  |  |
| <25 (n=162,107) | 1.00 (reference) | 1.12 (0.99, 1.27) | 1.05 (0.88, 1.26) | 1.49 (1.20, 1.86) | <0.01 | 0.28 |
| ≥25 (n=330,158) | 1.00 (reference) | 1.12 (1.04, 1.20) | 1.12 (1.02, 1.23) | 1.29 (1.13, 1.46) | <0.0001 |  |
| Abbreviation: MET, metabolic equivalent. | | | | | | |
| ^1^ Values are hazard ratios (95% confidence interval) unless otherwise indicated, calculated by Cox proportional hazards models. | | | | | | |
| ^2^ *P* for trend was calculated by using the frequency of adding salt to foods (never/rarely: 1, sometimes: 2, usually: 3, and always: 4) as an ordinal variable. | | | | | | |
| ^3^ *P* for interaction was calculated by adding interaction terms to the Cox models. | | | | | | |
| Adjusted for age, sex, ethnicity, Townsend deprivation index, smoking status, alcohol drinking status, physical activity, sedentary time, cardiovascular disease, cancer, diabetes, hyperlipidemia, hypertension, and healthy diet score. | | | | | | |

| **Supplementary Table 4.** Stratified analysis of associations of the frequency of adding salt to foods with risk of incident HCC ^1^ | | | | | | |
| --- | --- | --- | --- | --- | --- | --- |
| Subgroup | Frequency of adding salt to foods | | | | *P* for trend ^2^ | *P* for interaction ^3^ |
|  | Never/rarely | Sometimes | Usually | Always |  |  |
| **Age** (years) |  |  |  |  |  |  |
| <60 (n=278,848) | 1.00 (reference) | 0.82 (0.51, 1.31) | 1.50 (0.90, 2.51) | 1.9 (1.00, 3.63) | 0.0427 | 0.58 |
| ≥60 (n=213,417) | 1.00 (reference) | 1.53 (1.17, 1.99) | 1.53 (1.09, 2.14) | 2.56 (1.71, 3.84) | <0.0001 |  |
| **Sex** |  |  |  |  |  |  |
| Male (n=22,2870) | 1.00 (reference) | 1.51 (1.16, 1.98) | 1.61 (1.16, 2.23) | 2.82 (1.93, 4.13) | <0.0001 | 0.04 |
| Female (n=26,9395) | 1.00 (reference) | 0.83 (0.52, 1.34) | 1.36 (0.77, 2.41) | 1.19 (0.51, 2.80) | 0.51 |  |
| **Ethnicity** |  |  |  |  |  |  |
| White (n=464,104) | 1.00 (reference) | 1.24 (0.98, 1.57) | 1.52 (1.14, 2.03) | 2.35 (1.66, 3.34) | <0.0001 | 0.84 |
| Others (n=28,161) | 1.00 (reference) | 4.01 (1.08, 14.9) | 2.00 (0.33, 12.2) | 3.12 (0.51, 19.1) | 0.24 |  |
| **Townsend deprivation index** |  |  |  |  |  |  |
| <Median (n=245,760) | 1.00 (reference) | 1.67 (1.20, 2.32) | 1.66 (1.09, 2.55) | 2.42 (1.36, 4.31) | <0.001 | 0.91 |
| ≥Median (n=246,505) | 1.00 (reference) | 1.02 (0.74, 1.42) | 1.40 (0.96, 2.04) | 2.20 (1.43, 3.38) | <0.001 |  |
| **Smoking status** |  |  |  |  |  |  |
| Current (n=50,316) | 1.00 (reference) | 0.86 (0.47, 1.59) | 1.19 (0.62, 2.26) | 1.30 (0.62, 2.73) | 0.43 | 0.45 |
| Previous (n=169,705) | 1.00 (reference) | 1.32 (0.94, 1.85) | 1.65 (1.11, 2.44) | 2.45 (1.49, 4.03) | <0.001 |  |
| Never (n=270,375) | 1.00 (reference) | 1.47 (1.02, 2.13) | 1.32 (0.76, 2.27) | 3.13 (1.72, 5.73) | <0.01 |  |
| **Alcohol drinking status** |  |  |  |  |  |  |
| Current (n=452,706) | 1.00 (reference) | 1.38 (1.08, 1.75) | 1.55 (1.15, 2.08) | 2.42 (1.68, 3.48) | <0.0001 | 0.52 |
| Previous (n=16,955) | 1.00 (reference) | 0.60 (0.20, 1.84) | 1.00 (0.28, 3.58) | 1.48 (0.41, 5.42) | 0.76 |  |
| Never (n=22,073) | 1.00 (reference) | 0.85 (0.22, 3.25) | 1.27 (0.26, 6.17) | 2.37 (0.48, 11.8) | 0.39 |  |
| **Physical activity** (MET-minutes/week) |  |  |  |  |  |  |
| <600 (n=73,905) | 1.00 (reference) | 1.45 (0.83, 2.56) | 1.93 (1.00, 3.71) | 3.67 (1.82, 7.39) | <0.001 | 0.20 |
| ≥600 (n=418,360) | 1.00 (reference) | 1.27 (0.98, 1.63) | 1.44 (1.05, 1.97) | 2.04 (1.37, 3.04) | <0.001 |  |
| **Sedentary time** (hours/day) |  |  |  |  |  |  |
| <Median (n=235,418) | 1.00 (reference) | 1.54 (1.06, 2.24) | 1.50 (0.90, 2.49) | 3.78 (2.18, 6.56) | <0.0001 | 0.30 |
| ≥Median (n=256,847) | 1.00 (reference) | 1.18 (0.88, 1.58) | 1.51 (1.07, 2.12) | 1.82 (1.17, 2.81) | <0.01 |  |
| **Cardiovascular disease** |  |  |  |  |  |  |
| Yes (n=38,877) | 1.00 (reference) | 1.11 (0.63, 1.94) | 1.33 (0.68, 2.60) | 2.17 (1.01, 4.68) | 0.07 | 0.81 |
| No (n=453,388) | 1.00 (reference) | 1.34 (1.04, 1.73) | 1.56 (1.15, 2.14) | 2.37 (1.61, 3.48) | <0.0001 |  |
| **Cancer** |  |  |  |  |  |  |
| Yes (n=42,863) | 1.00 (reference) | 0.72 (0.30, 1.75) | 1.30 (0.51, 3.34) | 1.00 (0.22, 4.47) | 0.84 | 0.31 |
| No (n=449,402) | 1.00 (reference) | 1.36 (1.07, 1.73) | 1.55 (1.15, 2.08) | 2.49 (1.75, 3.55) | <0.0001 |  |
| **Diabetes** |  |  |  |  |  |  |
| Yes (n=29,809) | 1.00 (reference) | 1.20 (0.81, 1.77) | 1.37 (0.84, 2.25) | 2.04 (1.11, 3.75) | 0.02 | 0.39 |
| No (n=46,2456) | 1.00 (reference) | 1.35 (1.02, 1.80) | 1.60 (1.13, 2.26) | 2.51 (1.66, 3.80) | <0.0001 |  |
| **Hyperlipidemia** |  |  |  |  |  |  |
| Yes (n=395,421) | 1.00 (reference) | 1.30 (1.01, 1.66) | 1.54 (1.14, 2.09) | 2.27 (1.56, 3.32) | <0.0001 | 0.60 |
| No (n=96,844) | 1.00 (reference) | 1.34 (0.73, 2.45) | 1.39 (0.64, 3.00) | 2.80 (1.22, 6.39) | 0.03 |  |
| **Hypertension** |  |  |  |  |  |  |
| Yes (n=272,577) | 1.00 (reference) | 1.47 (1.14, 1.9) | 1.61 (1.18, 2.21) | 2.48 (1.68, 3.64) | <0.0001 | 0.51 |
| No (n=219,688) | 1.00 (reference) | 0.70 (0.39, 1.25) | 1.17 (0.62, 2.22) | 1.85 (0.88, 3.92) | 0.23 |  |
| **Healthy diet score** |  |  |  |  |  |  |
| <Median (n=159,058) | 1.00 (reference) | 1.06 (0.74, 1.53) | 1.55 (1.05, 2.30) | 2.24 (1.43, 3.52) | <0.001 | 0.47 |
| ≥Median (n=333,207) | 1.00 (reference) | 1.50 (1.12, 2.02) | 1.41 (0.93, 2.14) | 2.26 (1.31, 3.90) | <0.01 |  |
| **Body mass index** (kg/m^2^) |  |  |  |  |  |  |
| <25 (n=162,107) | 1.00 (reference) | 0.98 (0.54, 1.78) | 0.98 (0.43, 2.24) | 2.79 (1.28, 6.06) | 0.08 | 0.88 |
| ≥25 (n=330,158) | 1.00 (reference) | 1.35 (1.05, 1.74) | 1.60 (1.18, 2.17) | 2.23 (1.52, 3.26) | <0.0001 |  |
| Abbreviations: HCC, hepatocellular carcinoma; MET, metabolic equivalent. | | | | | | |
| ^1^ Values are hazard ratios (95% confidence interval) unless otherwise indicated, calculated by Cox proportional hazards models. | | | | | | |
| ^2^ *P* for trend was calculated by using the frequency of adding salt to foods (never/rarely: 1, sometimes: 2, usually: 3, and always: 4) as an ordinal variable. | | | | | | |
| ^3^ *P* for interaction was calculated by adding interaction terms to the Cox models. | | | | | | |
| Adjusted for age, sex, ethnicity, Townsend deprivation index, smoking status, alcohol drinking status, physical activity, sedentary time, cardiovascular disease, cancer, diabetes, hyperlipidemia, hypertension, and healthy diet score. | | | | | | |

| **Supplementary Table 5.** Associations of the frequency of adding salt to foods with risk of incident MASLD, cirrhosis, and HCC, excluding the outcomes of interest that occurred within the first two years of follow-up ^1^ | | | | | |
| --- | --- | --- | --- | --- | --- |
|  | Frequency of adding salt to foods | | | | *P* for trend ^2^ |
|  | Never/rarely | Sometimes | Usually | Always |  |
| **MASLD** |  |  |  |  |  |
| Number of participants | 273,665 | 137,986 | 56,941 | 23,388 |  |
| Number of cases | 3,358 | 1,936 | 933 | 493 |  |
| Person-years | 3,677,716 | 1,849,754 | 759,107 | 309,068 |  |
| Incidence per 1000 person-years | 0.91 | 1.05 | 1.23 | 1.60 |  |
| Model 1 | 1.00 (reference) | 1.12 (1.06, 1.18) | 1.28 (1.19, 1.38) | 1.57 (1.42, 1.72) | <0.0001 |
| Model 2 | 1.00 (reference) | 1.10 (1.04, 1.16) | 1.25 (1.16, 1.35) | 1.43 (1.30, 1.58) | <0.0001 |
| Model 3 | 1.00 (reference) | 1.09 (1.03, 1.15) | 1.22 (1.14, 1.32) | 1.38 (1.26, 1.52) | <0.0001 |
| Model 4 | 1.00 (reference) | 1.04 (0.99, 1.11) | 1.17 (1.08, 1.25) | 1.32 (1.20, 1.46) | <0.0001 |
| **Cirrhosis** |  |  |  |  |  |
| Number of participants | 273,600 | 137,925 | 56,922 | 23,388 |  |
| Number of cases | 2,592 | 1514 | 665 | 345 |  |
| Person-years | 3,684,216 | 1,853,412 | 760,987 | 310,155 |  |
| Incidence per 1000 person-years | 0.70 | 0.82 | 0.87 | 1.11 |  |
| Model 1 | 1.00 (reference) | 1.14 (1.07, 1.22) | 1.17 (1.07, 1.27) | 1.50 (1.34, 1.68) | <0.0001 |
| Model 2 | 1.00 (reference) | 1.12 (1.05, 1.20) | 1.13 (1.04, 1.23) | 1.38 (1.23, 1.55) | <0.0001 |
| Model 3 | 1.00 (reference) | 1.11 (1.04, 1.19) | 1.11 (1.02, 1.21) | 1.34 (1.20, 1.51) | <0.0001 |
| Model 4 | 1.00 (reference) | 1.10 (1.03, 1.17) | 1.09 (1.00, 1.19) | 1.32 (1.18, 1.48) | <0.0001 |
| **HCC** |  |  |  |  |  |
| Number of participants | 273,805 | 138,060 | 56,975 | 23,412 |  |
| Number of cases | 173 | 119 | 68 | 40 |  |
| Person-years | 3,691,841 | 1,858,038 | 762,987 | 311,178 |  |
| Incidence per 1000 person-years | 0.05 | 0.06 | 0.09 | 0.13 |  |
| Model 1 | 1.00 (reference) | 1.33 (1.06, 1.69) | 1.64 (1.24, 2.17) | 2.55 (1.80, 3.60) | <0.0001 |
| Model 2 | 1.00 (reference) | 1.30 (1.03, 1.64) | 1.60 (1.20, 2.12) | 2.38 (1.68, 3.38) | <0.0001 |
| Model 3 | 1.00 (reference) | 1.27 (1.01, 1.61) | 1.53 (1.15, 2.04) | 2.23 (1.57, 3.18) | <0.0001 |
| Model 4 | 1.00 (reference) | 1.23 (0.98, 1.56) | 1.47 (1.10, 1.95) | 2.14 (1.50, 3.04) | <0.0001 |
| Abbreviations: HCC, hepatocellular carcinoma; MASLD, metabolic dysfunction-associated steatotic liver disease. | | | | | |
| ^1^ Values are hazard ratios (95% confidence interval) unless otherwise indicated, calculated by Cox proportional hazards models. | | | | | |
| ^2^ *P* for trend was calculated by using the frequency of adding salt to foods (never/rarely: 1, sometimes: 2, usually: 3, and always: 4) as an ordinal variable. | | | | | |
| Model 1 was adjusted for age, sex, ethnicity, and Townsend deprivation index. | | | | | |
| Model 2 was further adjusted for smoking status, alcohol drinking status, physical activity, sedentary time, cardiovascular disease, cancer, diabetes, hyperlipidemia, and hypertension. | | | | | |
| Model 3 was additionally adjusted for healthy diet score. | | | | | |
| Model 4 was additionally adjusted for body mass index. | | | | | |

| **Supplementary Table 6.** Associations of the frequency of adding salt to foods with risk of incident MASLD, cirrhosis, and HCC, excluding participants who had major dietary changes in the last five years ^1^ | | | | | |
| --- | --- | --- | --- | --- | --- |
|  | Frequency of adding salt to foods | | | | *P* for trend ^2^ |
|  | Never/rarely | Sometimes | Usually | Always |  |
| Number of participants | 165,351 | 84,080 | 35,660 | 14,999 |  |
| **MASLD** |  |  |  |  |  |
| Number of cases | 1,620 | 908 | 483 | 284 |  |
| Person-years | 2,227,770 | 1,130,349 | 476,620 | 198,648 |  |
| Incidence per 1000 person-years | 0.73 | 0.80 | 1.01 | 1.43 |  |
| Model 1 | 1.00 (reference) | 1.08 (0.99, 1.17) | 1.32 (1.19, 1.46) | 1.76 (1.55, 1.99) | <0.0001 |
| Model 2 | 1.00 (reference) | 1.05 (0.96, 1.14) | 1.24 (1.12, 1.37) | 1.51 (1.33, 1.72) | <0.0001 |
| Model 3 | 1.00 (reference) | 1.03 (0.95, 1.11) | 1.19 (1.07, 1.32) | 1.42 (1.25, 1.62) | <0.0001 |
| Model 4 | 1.00 (reference) | 0.99 (0.91, 1.07) | 1.13 (1.02, 1.26) | 1.36 (1.19, 1.54) | <0.0001 |
| **Cirrhosis** |  |  |  |  |  |
| Number of cases | 1,529 | 913 | 400 | 234 |  |
| Person-years | 2,230,027 | 1,131,542 | 477,505 | 199,042 |  |
| Incidence per 1000 person-years | 0.69 | 0.81 | 0.84 | 1.18 |  |
| Model 1 | 1.00 (reference) | 1.16 (1.07, 1.26) | 1.14 (1.02, 1.27) | 1.60 (1.39, 1.84) | <0.0001 |
| Model 2 | 1.00 (reference) | 1.13 (1.04, 1.23) | 1.08 (0.96, 1.20) | 1.42 (1.24, 1.64) | <0.0001 |
| Model 3 | 1.00 (reference) | 1.12 (1.03, 1.21) | 1.05 (0.94, 1.18) | 1.38 (1.20, 1.59) | <0.001 |
| Model 4 | 1.00 (reference) | 1.10 (1.02, 1.20) | 1.04 (0.93, 1.16) | 1.36 (1.18, 1.57) | <0.001 |
| **HCC** |  |  |  |  |  |
| Number of cases | 85 | 64 | 42 | 27 |  |
| Person-years | 2,233,801 | 1,133,916 | 478,465 | 199,690 |  |
| Incidence per 1000 person-years | 0.04 | 0.06 | 0.09 | 0.14 |  |
| Model 1 | 1.00 (reference) | 1.44 (1.04, 1.99) | 1.96 (1.35, 2.83) | 3.14 (2.03, 4.87) | <0.0001 |
| Model 2 | 1.00 (reference) | 1.39 (1.01, 1.93) | 1.85 (1.27, 2.69) | 2.80 (1.80, 4.37) | <0.0001 |
| Model 3 | 1.00 (reference) | 1.38 (1.00, 1.91) | 1.82 (1.25, 2.65) | 2.73 (1.74, 4.27) | <0.0001 |
| Model 4 | 1.00 (reference) | 1.35 (0.97, 1.86) | 1.74 (1.20, 2.54) | 2.64 (1.69, 4.13) | <0.0001 |
| Abbreviations: HCC, hepatocellular carcinoma; MASLD, metabolic dysfunction-associated steatotic liver disease. | | | | | |
| ^1^ Values are hazard ratios (95% confidence interval) unless otherwise indicated, calculated by Cox proportional hazards models. | | | | | |
| ^2^ *P* for trend was calculated by using the frequency of adding salt to foods (never/rarely: 1, sometimes: 2, usually: 3, and always: 4) as an ordinal variable. | | | | | |
| Model 1 was adjusted for age, sex, ethnicity, and Townsend deprivation index. | | | | | |
| Model 2 was further adjusted for smoking status, alcohol drinking status, physical activity, sedentary time, cardiovascular diseases, cancer, diabetes, hyperlipidemia, and hypertension. | | | | | |
| Model 3 was additionally adjusted for healthy diet score. | | | | | |
| Model 4 was additionally adjusted for body mass index. | | | | | |

| **Supplementary Table 7.** Associations of the frequency of adding salt to foods with risk of incident MASLD, cirrhosis, and hepatocellular carcinoma, excluding MASLD cases with significant alcohol intake and adjusting for alcohol intake instead of alcohol drinking status ^1^ | | | | | |
| --- | --- | --- | --- | --- | --- |
|  | Frequency of adding salt to foods | | | | *P* for trend ^2^ |
|  | Never/rarely | Sometimes | Usually | Always |  |
| Number of participants | 273,088 | 137,564 | 56,683 | 23,259 |  |
| **MASLD** |  |  |  |  |  |
| Number of cases | 2,781 | 1,514 | 675 | 364 |  |
| Person-years | 3,671,012 | 1,845,184 | 756,283 | 307,605 |  |
| Incidence per 1000 person-years | 0.76 | 0.82 | 0.89 | 1.18 |  |
| Model 1 | 1.00 (reference) | 1.05 (0.99, 1.12) | 1.13 (1.04, 1.23) | 1.36 (1.22, 1.52) | <0.0001 |
| Model 2 | 1.00 (reference) | 1.06 (1.00, 1.13) | 1.19 (1.10, 1.30) | 1.42 (1.27, 1.59) | <0.0001 |
| Model 3 | 1.00 (reference) | 1.05 (0.99, 1.12) | 1.17 (1.08, 1.28) | 1.38 (1.24, 1.55) | <0.0001 |
| Model 4 | 1.00 (reference) | 1.01 (0.95, 1.08) | 1.11 (1.02, 1.20) | 1.30 (1.17, 1.46) | <0.0001 |
| **Cirrhosis** |  |  |  |  |  |
| Number of cases | 2,695 | 1571 | 679 | 335 |  |
| Person-years | 3,675,218 | 1,847,256 | 757,264 | 308,271 |  |
| Incidence per 1000 person-years | 0.73 | 0.85 | 0.90 | 1.09 |  |
| Model 1 | 1.00 (reference) | 1.14 (1.08, 1.22) | 1.15 (1.06, 1.25) | 1.41 (1.26, 1.58) | <0.0001 |
| Model 2 | 1.00 (reference) | 1.11 (1.04, 1.18) | 1.09 (1.00, 1.18) | 1.24 (1.10, 1.39) | <0.0001 |
| Model 3 | 1.00 (reference) | 1.11 (1.04, 1.18) | 1.07 (0.98, 1.17) | 1.21 (1.08, 1.36) | <0.001 |
| Model 4 | 1.00 (reference) | 1.09 (1.02, 1.16) | 1.05 (0.97, 1.15) | 1.19 (1.06, 1.34) | <0.01 |
| **Hepatocellular carcinoma** |  |  |  |  |  |
| Number of cases | 164 | 116 | 66 | 39 |  |
| Person-years | 3,682,262 | 1,851,414 | 759,048 | 309,158 |  |
| Incidence per 1000 person-years | 0.04 | 0.06 | 0.09 | 0.13 |  |
| Model 1 | 1.00 (reference) | 1.37 (1.08, 1.74) | 1.69 (1.27, 2.25) | 2.63 (1.85, 3.73) | <0.0001 |
| Model 2 | 1.00 (reference) | 1.31 (1.03, 1.67) | 1.56 (1.17, 2.09) | 2.22 (1.55, 3.17) | <0.0001 |
| Model 3 | 1.00 (reference) | 1.29 (1.02, 1.64) | 1.52 (1.14, 2.03) | 2.12 (1.48, 3.04) | <0.0001 |
| Model 4 | 1.00 (reference) | 1.26 (0.99, 1.6) | 1.46 (1.09, 1.95) | 2.05 (1.43, 2.94) | <0.0001 |
| Abbreviation: MASLD, metabolic dysfunction-associated steatotic liver disease. | | | | | |
| ^1^ Values are hazard ratios (95% confidence interval) unless otherwise indicated, calculated by Cox proportional hazards models. | | | | | |
| ^2^ *P* for trend was calculated by using the frequency of adding salt to foods (never/rarely: 1, sometimes: 2, usually: 3, and always: 4) as an ordinal variable. | | | | | |
| Model 1 was adjusted for age, sex, ethnicity, and Townsend deprivation index. | | | | | |
| Model 2 was further adjusted for smoking status, alcohol intake, physical activity, sedentary time, cardiovascular diseases, cancer, diabetes, hyperlipidemia, and hypertension. | | | | | |
| Model 3 was additionally adjusted for healthy diet score. | | | | | |
| Model 4 was additionally adjusted for body mass index. | | | | | |

| **Supplementary Table 8.** Associations of the frequency of adding salt to foods with risk of incident MASLD, cirrhosis, and HCC, adjusting for dietary intake collected from 24-hour recall ^1^ | | | | | |  |
| --- | --- | --- | --- | --- | --- | --- |
|  | Frequency of adding salt to foods | | | | *P* for trend ^2^ |  |
|  | Never/rarely | Sometimes | Usually | Always |  |  |
| Number of participants | 122,383 | 56,237 | 22,213 | 7,089 |  |  |
| **MASLD** |  |  |  |  |  |  |
| Number of cases | 1,222 | 675 | 340 | 125 |  |  |
| Person-years | 1,629,440 | 746,346 | 293,276 | 93,030 |  |  |
| Incidence per 1000 person-years | 0.75 | 0.90 | 1.16 | 1.34 |  |  |
| Model 1 | 1.00 (reference) | 1.18 (1.08, 1.30) | 1.48 (1.31, 1.67) | 1.67 (1.39, 2.01) | <0.0001 |  |
| Model 2 | 1.00 (reference) | 1.16 (1.06, 1.28) | 1.42 (1.25, 1.60) | 1.50 (1.24, 1.80) | <0.0001 |  |
| Model 3 | 1.00 (reference) | 1.14 (1.04, 1.26) | 1.38 (1.22, 1.56) | 1.42 (1.17, 1.71) | <0.0001 |  |
| Model 4 | 1.00 (reference) | 1.10 (1.00, 1.21) | 1.31 (1.16, 1.48) | 1.33 (1.11, 1.61) |  |  |
| **Cirrhosis** |  |  |  |  |  |  |
| Number of cases | 1,011 | 539 | 221 | 82 |  |  |
| Person-years | 1,631,574 | 747,517 | 294,086 | 93,310 |  |  |
| Incidence per 1000 person-years | 0.62 | 0.72 | 0.75 | 0.88 |  |  |
| Model 1 | 1.00 (reference) | 1.15 (1.04, 1.28) | 1.15 (0.99, 1.33) | 1.40 (1.12, 1.75) | <0.001 |  |
| Model 2 | 1.00 (reference) | 1.14 (1.02, 1.26) | 1.11 (0.96, 1.28) | 1.29 (1.03, 1.62) | <0.01 |  |
| Model 3 | 1.00 (reference) | 1.13 (1.01, 1.25) | 1.09 (0.94, 1.26) | 1.24 (0.98, 1.55) | 0.02 |  |
| Model 4 | 1.00 (reference) | 1.11 (1.00, 1.24) | 1.07 (0.92, 1.24) | 1.22 (0.97, 1.53) | 0.04 |  |
| **HCC** |  |  |  |  |  |  |
| Number of cases | 53 | 45 | 18 | 9 |  |  |
| Person-years | 1,634,225 | 749,076 | 294,637 | 93,536 |  |  |
| Incidence per 1000 person-years | 0.03 | 0.06 | 0.06 | 0.10 |  |  |
| Model 1 | 1.00 (reference) | 1.82 (1.23, 2.71) | 1.67 (0.98, 2.85) | 2.95 (1.45, 5.99) | <0.001 |  |
| Model 2 | 1.00 (reference) | 1.83 (1.23, 2.72) | 1.66 (0.97, 2.85) | 2.95 (1.45, 6.03) | <0.001 |  |
| Model 3 | 1.00 (reference) | 1.73 (1.16, 2.59) | 1.52 (0.88, 2.61) | 2.57 (1.25, 5.27) | <0.01 |  |
| Model 4 | 1.00 (reference) | 1.67 (1.12, 2.49) | 1.45 (0.84, 2.48) | 2.45 (1.20, 5.02) | <0.01 |  |
| Abbreviations: HCC, hepatocellular carcinoma; MASLD, metabolic dysfunction-associated steatotic liver disease. | | | | | |  |
| ^1^ Values are hazard ratios (95% confidence interval) unless otherwise indicated, calculated by Cox proportional hazards models. | | | | | |  |
| ^2^ *P* for trend was calculated by using the frequency of adding salt to foods (never/rarely: 1, sometimes: 2, usually: 3, and always: 4) as an ordinal variable. | | | | | |  |
| Model 1 was adjusted for age, sex, ethnicity, and Townsend deprivation index. | | | | | |  |
| Model 2 was further adjusted for smoking status, alcohol drinking status, physical activity, sedentary time, cardiovascular disease, cancer, diabetes, hyperlipidemia, and hypertension. | | | | | |  |
| Model 3 was additionally adjusted for fruits, vegetables, red and processed meats, fish, sugar-sweetened beverages, and total energy intake. | | | | | |  |
| Model 4 was additionally adjusted for body mass index. | | | | | |  |

| **Supplementary Table 9.** Associations of the frequency of adding salt to foods with sodium and potassium in urine ^1^ | | | | | |
| --- | --- | --- | --- | --- | --- |
|  | Frequency of adding salt to foods | | | | *P* for trend ^2^ |
|  | Never/rarely | Sometimes | Usually | Always |  |
| Number of participants | 262,295 | 132,415 | 54,499 | 22,218 |  |
| Spot urinary sodium (mmol/L) | 71.0 (70.0, 72.0) | 76.1 (75.1, 77.2) | 79.3 (78.1, 80.4) | 83.1 (81.8, 84.4) | <0.0001 |
| Estimated 24-hour sodium excretion (g/day) | 3.31 (3.30, 3.33) | 3.38 (3.36, 3.39) | 3.41 (3.40, 3.43) | 3.45 (3.44, 3.47) | <0.0001 |
| ^1^ Values are least square geometric means (95% confidence intervals) unless otherwise indicated, calculated by general linear models. | | | | | |
| ^2^ *P* for trend was calculated by using the frequency of adding salt to foods (never/rarely: 1, sometimes: 2, usually: 3, and always: 4) as an ordinal variable. | | | | | |
| Adjusted for age, sex, ethnicity, Townsend deprivation index, smoking status, alcohol drinking status, physical activity, sedentary time, cardiovascular disease, cancer, diabetes, hyperlipidemia, hypertension, healthy diet score, body mass index, and spot urinary potassium (only for urinary sodium analysis). For the estimated 24-hour sodium excretion analysis, age and body mass index were not adjusted in the multivariable model because the sex-specific INTERSALT formulae included age and body mass index. | | | | | |

| **Supplementary Table 10.** Associations of sodium in urine with risk of incident MASLD, cirrhosis, and HCC ^1^ | | | | | |
| --- | --- | --- | --- | --- | --- |
|  | Quartile 1 | Quartile 2 | Quartile 3 | Quartile 4 | *P* for trend ^2^ |
| **Spot urinary sodium** |  |  |  |  |  |
| Number of participants | 117,922 | 118,166 | 117,281 | 118,058 |  |
| MASLD | 1.00 (reference) | 1.10 (1.01, 1.19) | 1.10 (1.02, 1.19) | 1.18 (1.09, 1.28) | <0.001 |
| Cirrhosis | 1.00 (reference) | 0.91 (0.84, 0.99) | 0.85 (0.79, 0.93) | 0.82 (0.75, 0.90) | <0.0001 |
| HCC | 1.00 (reference) | 1.00 (0.73, 1.37) | 0.96 (0.70, 1.32) | 0.90 (0.65, 1.25) | 0.46 |
| **Estimated 24-hour sodium excretion** |  |  |  |  |  |
| Number of participants | 117,857 | 117,857 | 117,856 | 117,857 |  |
| MASLD | 1.00 (reference) | 1.54 (1.42, 1.67) | 2.25 (2.07, 2.45) | 3.36 (3.04, 3.71) | <0.0001 |
| Cirrhosis | 1.00 (reference) | 0.81 (0.75, 0.88) | 0.85 (0.77, 0.94) | 0.93 (0.83, 1.04) | 0.51 |
| HCC | 1.00 (reference) | 0.82 (0.55, 1.22) | 0.83 (0.55, 1.27) | 1.03 (0.67, 1.60) | 0.92 |
| Abbreviations: HCC, hepatocellular carcinoma; MASLD, metabolic dysfunction-associated steatotic liver disease. | | | | | |
| ^1^ Values are hazard ratios (95% confidence interval) unless otherwise indicated, calculated by Cox proportional hazards models. | | | | | |
| ^2^ *P* for trend was calculated by fitting the median value of each quartile as a continuous variable. | | | | | |
| ^1^ Adjusted for age, sex, ethnicity, Townsend deprivation index, smoking status, alcohol drinking status, physical activity, sedentary time, cardiovascular diseases, cancer, diabetes, hyperlipidemia, hypertension, healthy diet score, body mass index, and spot urinary potassium. For the estimated 24-hour sodium excretion analysis, age and body mass index were not adjusted in the multivariable model because the sex-specific INTERSALT formulae included age and body mass index. | | | | | |

| **Supplementary Table 11.** Association between the frequency of adding salt to foods with MASLD, defined by magnetic resonance imaging proton density fat fraction ^1^ | | | | | |
| --- | --- | --- | --- | --- | --- |
|  | Frequency of adding salt to foods | | | | *P* for trend ^2^ |
|  | Never/rarely | Sometimes | Usually | Always |  |
| Number of participants | 24,359 | 10,736 | 4,192 | 1,204 |  |
| Number of cases | 6,252 | 3,201 | 1,332 | 397 |  |
| Model 1 | 1.00 (reference) | 1.22 (1.16, 1.29) | 1.30 (1.21, 1.40) | 1.43 (1.26, 1.62) | <0.0001 |
| Model 2 | 1.00 (reference) | 1.21 (1.14, 1.27) | 1.27 (1.18, 1.37) | 1.30 (1.14, 1.48) | <0.0001 |
| Model 3 | 1.00 (reference) | 1.18 (1.12, 1.25) | 1.23 (1.14, 1.33) | 1.24 (1.09, 1.42) | <0.0001 |
| Model 4 | 1.00 (reference) | 1.14 (1.07, 1.20) | 1.15 (1.07, 1.25) | 1.13 (0.99, 1.30) | <0.0001 |
| Abbreviation: MASLD, metabolic dysfunction-associated steatotic liver disease. | | | | | |
| ^1^ Values are odds ratios (95% confidence interval) unless otherwise indicated, calculated by logistic models. | | | | | |
| ^2^ *P* for trend was calculated by using the frequency of adding salt to foods (never/rarely: 1, sometimes: 2, usually: 3, and always: 4) as an ordinal variable. | | | | | |
| Model 1 was adjusted for age, sex, ethnicity, and Townsend deprivation index. | | | | | |
| Model 2 was further adjusted for smoking status, alcohol drinking status, physical activity, sedentary time, cardiovascular diseases, cancer, diabetes, hyperlipidemia, and hypertension. | | | | | |
| Model 3 was additionally adjusted for healthy diet score. | | | | | |
| Model 4 was additionally adjusted for body mass index. | | | | | |

| **Supplementary Table 12.** Mediation analyses of diabetes and alcohol intake on the associations of the frequency of adding salt to foods with the risk of incident MASLD, cirrhosis, and HCC | | | | |  |
| --- | --- | --- | --- | --- | --- |
|  | Hazard ratio (95% confidence interval) | | Proportion mediated | *P* value |  |
|  | Unadjusted for the factor | Adjusted for the factor |  |  |  |
| **MASLD** |  |  |  |  |  |
| Diabetes | 1.10 (1.07, 1.13) | 1.11 (1.08, 1.14) | - | - |  |
| Alcohol intake | 1.06 (1.03, 1.10) | 1.10 (1.06, 1.13) | - | - |  |
| **Cirrhosis** |  |  |  |  |  |
| Diabetes | 1.08 (1.05, 1.11) | 1.08 (1.05, 1.12) | - | - |  |
| Alcohol intake | 1.07 (1.04, 1.10) | 1.06 (1.03, 1.09) | 19.5% (11.8%, 30.5%) | <0.0001 |  |
| HCC |  |  |  |  |  |
| Diabetes | 1.28 (1.15, 1.42) | 1.30 (1.17, 1.44) | - | - |  |
| Alcohol intake | 1.29 (1.16, 1.43) | 1.26 (1.14, 1.40) | 8.2% (4.5%, 14.4%) | <0.0001 |  |
| Abbreviations: HCC, hepatocellular carcinoma; MASLD, metabolic dysfunction-associated steatotic liver disease. | | | | |  |
| For diabetes analyses, Cox models were adjusted for age, sex, ethnicity, Townsend deprivation index, smoking status, alcohol drinking status, physical activity, sedentary time, cardiovascular diseases, cancer, hyperlipidemia, hypertension, and healthy diet score. | | | | |  |
| For alcohol intake analyses, MASLD cases with significant alcohol intake were excluded, and Cox models were adjusted for age, sex, ethnicity, Townsend deprivation index, smoking status, physical activity, sedentary time, cardiovascular diseases, cancer, diabetes, hyperlipidemia, hypertension, and healthy diet score. | | | | |  |
| Because the transform the macro uses to get the confidence interval for the proportion mediated does not go below zero, the confidence interval does not contain the null value (0) but a non-significant *P* value. Pay attention to the *P* value, not the confidence interval. | | | | |  |
